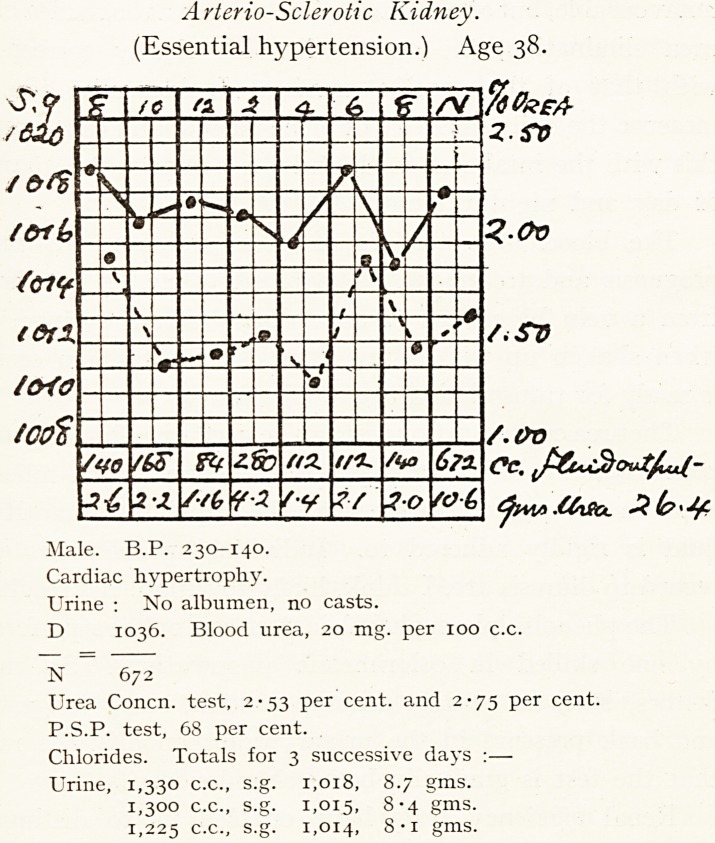# Discussion on Renal Efficiency

**Published:** 1922-06

**Authors:** Cecil Clarke, J. O. Symes, G. Hadfield

**Affiliations:** Assistant Physician to the Bristol General Hospital; Physician to the Bristol General Hospital; Pathologist to the Bristol General Hospital


### RENAL EFFICIENCY.

A Discussion held at a meeting of the Bristol Medico-Chirurgical Society on November 9th, 1921.

---

Cecil Clarke, M.D. Lond., M.R.C.P.,
*Assistant Physician to the Bristol General Hospital.*

The modern methods employed in the investigation of the renal efficiency are directed towards an estimation of those substances which, if the blood plasma is to remain of constant chemical composition, must be excreted. In actual practice this means the non-protein nitrogenous substances (chiefly urea) the chlorides and water.

Two other functions of the kidney, the maintenance of the normal osmotic pressure of the blood and of the normal reaction of the blood, still remain as regard their application to clinical medicine largely in the province of the experimental physiologist.

No single test can do more than give an indication of the depressed function of the kidney at the time the test is performed, nor can it indicate the possibility of functional repair ; and, further, it cannot be emphasised too strongly that the interpretation of any tests is impossible apart from a consideration of the clinical state, more especially of the cardio-vascular system.

It may be well to recall some of the accepted facts of the normal physiology of the kidney. In health the kidneys are only partially active. It has been estimated that one-fourth to one-sixth of the total kidney substance is sufficient for the average daily requirements. This very considerable reserve of potential activity in the normal meets all demands. On the experimental side Rose Bradford showed that large amounts of renal tissue, up to two-thirds of the total amount, could be removed and the animal kept in health.

The diseased kidney, on the other hand, works at a maximum, and thereby may maintain tolerable health for an indefinite period. By the time we obtain, in cases of chronic interstitial nephritis, a result suggesting exhaustion of this reserve a very considerable fraction of kidney substance, about three-quarters, has been destroyed.

From this time urea and other nitrogenous substances, such as uric acid, creatinin, amino-acids, and ammonia, tend to accumulate in the blood. Temporary circulatory changes in the kidney, *e.g.* passive congestion, inflammatory œdema, back pressure from mechanical obstruction to the outflow of urine, may produce a similar result indicative of partial or complete renal inefficiency.

Taken in conjunction with the clinical examination, the tests afford data for prognosis and treatment.

Dr. Symes and I have carried out a variety of renal efficiency tests in 50 cases of kidney disease, and have learned to place the greatest reliance on those concerned with the capacity of the kidney to excrete urea, namely the two-hour specific gravity test of Mosenthal, the urea concentration test, and the estimation of the blood urea.

*Two-Hour Test.*

There are certain details which require emphasis. The amount of fluid given should be known and should be at least 2,000 c.c., the diet should contain a moderately high amount of protein, the midday meal in particular should include about 5–10 grams of salt. Nothing is to be taken between meals, and the last day collection should be $2\frac{1}{2}$ hours after supper. The normal response shows a specific gravity varying more than eight or nine points, fluid taken at meals is quickly excreted, the day amount greater than the night, the latter not over 400 to 500 c.c. of a specific gravity of 1,018 or more. The day and night ratio is in health about 4 : 1 ; in nephritis it may alter to ratio $1\frac{1}{2}$ : 1 or 1 : 1. The total urine excreted should not be less than 75 per cent. of the fluid intake. The concentration of salt in the specimens varies considerably ; it is eliminated quickly, nitrogen more slowly.

Lichtwitz[1] carries out this procedure in much greater detail. First the normal day, then on following days three-quarters of 1 litre of water, 10 grams of sodium chloride, 20 grams of urea are added to his standard diet.

The important point for consideration is the fixation of the specific gravity : the level at which it is fixed depends on many factors.

Fixation at a low level is the usual finding in chronic interstitial nephritis, with an increase in the fluid output and nycturia (10 cases) ; fixation at a higher level in acute and subacute nephritis, most cases of parenchymatous nephritis, in the passive congestion of myocardial inefficiency. In one such case examined seven successive specimens were of specific gravity 1,016. In the arterio-sclerotic kidney the specific gravity is often fixed at a high level, but the results are variable, depending mainly on the circulatory efficiency.

A study of the normal response seems to show that the point on which stress is laid, namely a variation of more than eight points, depends chiefly on the excretion of urea and water primarily, chlorides to a less extent. The percentage of urea in the charts follows the specific gravity very closely.*

It is of interest to note that even in cases of diabetes mellitus undergoing the preliminary alimentary rest of modern methods of treatment one specimen yields a specific gravity of 1,020 (in the absence of dextrose). This is stated to occur in professional starvation.

Mosenthal's [2] observations on two points in treatment may be mentioned here. In the absence of disease a high fixed specific gravity is suggestive of too little intake of fluid; the resulting constipation and burning micturition require an increased intake of fluid by way of treatment.

In 6 cases of slight renal deficiency he found that a high protein intake was followed by a doubling of the night output.

Newburgh [3] noted that forced meat feeding in normal men caused the appearance of red blood cells in the urine. In 4 cases of high blood pressure such high protein diet was followed by a microscopic hæmaturia and the appearance or increase of albumin in the urine.

A disturbance in the fluid output is an early sign of damaged kidney. This is better ascertained from observing the relations between the day and night output than by the water excretion test; the latter test has given us very variable results.

*Urea Concentration Test.*

This test is easily carried out, and is perhaps the most widely accepted of all the renal tests. Its principle depends

* Note.—A 1% solution of Urea has a specific gravity of 1002·8.<br>

2% ,, ,, ,, ,, 1005·6.<br>

1% ,, NaCl ,, ,, 1007·2.<br>

A mixture of 2% Urea and 1% NaCl has a specific gravity of 1013.

Up to 7 parts per 1,000 albumen has very little influence on the specific gravity. The correction factor is 0·26 per 1%.

Specific gravity is taken with a *pyknometer*; urine at room temperature.

on the power of the kidney to concentrate urea; if the percentage of urea in the blood be taken as 0·02 per cent. and the urine contains 2 per cent., then the urea has been concentrated 100 times.

We have used the dose recommended by H. MacLean, namely 15 grams. McJunkin gives 30 grams, Lichtwitz 20 grams. An excretion of 2 per cent. or more means an efficient kidney. A low percentage, 1·4 per cent. or 1·5 per cent., suggests moderately severe renal inefficiency (MacLean). By the time we get a low result in chronic interstitial nephritis we can assume that a considerable fraction of the kidney substance has disappeared and that a terminal stage is approaching. In our 12 cases of chronic interstitial nephritis the low reading was associated with a rise in the blood urea figure in 9 out of 10 cases so examined.

MacLean,[4] summarising his experience of this test in 10,000 cases, points out one advantage, in that it indicates lesions of a much slighter degree than are detected by blood urea examinations. In cases of acute nephritis (MacLean) the urea test showed a defect long after the blood urea had gone back to normal. But we still require a test in clinical medicine which will indicate that a destruction of glomeruli is proceeding even when the kidney can furnish a specimen of urine containing 2 per cent. or more of urea. The only abnormal finding in a case of high blood pressure (Chart 4) was fixation of the specific gravity at a high level and nycturia.

At present this test is very widely used, and based on its results, the Ministry of Pensions have worked out a mathematical scale of awards, the pension awarded being inversely proportional to the amount of urea excreted. One caution is necessary—the urea occasionally causes a marked diuresis in those who have been imbibing large quantities of fluid prior to the dose. This diuresis may be sufficient to give a lower reading than normal. The normal response at the end of the second hour is from 80 to 150 c.c. of urine.

In 5 cases of chronic parenchymatous nephritis percentages of 2 or more were found. This remarkable result is difficult of explanation, but the gross albuminuria, the œdema and diminished chloride content of the urine, etc., are sufficient in themselves to indicate the severity of the renal condition.

*Blood Urea.*

The normal limits given by various writers vary from 10 to 25 milligrams per 100 c.c. of blood. There is general agreement that a figure above 40 mg. per 100 c.c. indicates a retention of urea in the blood.

The total non-protein nitrogenous substances and other extractives rise *pari passu* with urea nitrogen, and practically it is sufficient, and certainly easier, to determine the urea figure. Myers and Killian consider that the blood creatinin figure gives a more reliable figure for prognosis (normal 0·1 to 0·8 mg. per 100 c.c.; in renal disease from 2 to 42 mg.).

In acute nephritis the blood urea figure is of value, and although raised, it does not reach such high grades as in renal cirrhosis. In 6 of our cases of acute and subacute nephritis values of forty to sixty coincided with low values for urea excretion and with a severe clinical condition of nephritis. Lower values were associated with a steadily improving general condition. In chronic nephritis as above stated much higher values are found, 90 to 120 or more. Such figures indicate a definite gravity of prognosis. A rising quantity is undoubtedly of grave import. In one case the figure rose from 60 to 112 in an interval of six months. The inefficiency associated with myocardial failure may give also readings up to 180.

All cases of interstitial nephritis do not of necessity show an increase in the blood urea nitrogen. Up to a certain stage the excretion of urea may be the same as that of a healthy man. The lack of power of concentration of urea is met by an increased excretion of fluid, the damaged kidneys working at their utmost capacity, producing a constant output with a low urea value—a compensatory phenomenon.

Blood urea estimations have proved of value in cases of prostatic enlargement. A recent paper by Dobson [5] endorses this examination as the most valuable pre-operative prognostic test. The renal inefficiency consequent on back pressure effects is associated with a rise in the blood urea, a decided fall occurring after preliminary cystotomy in cases likely to do well. MacAdam [6] finds in such cases (number unspecified) a rise in the blood urea to 80–200 mg. per 100 c.c. After supra-pubic drainage for 7–14 days the figure fell to 20–30 mg. per 100 c.c. He considered that the mechanical back pressure produced its effect by interference with the normal filtration through the glomeruli, with the resultant nitrogen retention. Over 30 mg. per 100 c.c. was considered as a figure indicative of the necessity of preliminary treatment.

The blood count in 3 cases so examined of chronic interstitial nephritis with nitrogen retention showed a secondary anæmia :—

F., æt. 43, 105 mg. urea. Total reds, 3,075,000 per cm. Hb. 50 per cent.

M., æt. 48, 121 mg. urea. Total reds 3,500,000 per cm. Hb. 41 per cent.

F., æt. 56, 95 mg. urea. Total reds, 4,000,000 per cm. Hb. 55 per cent.

*Essential Hypertension* (arterio-sclerotic kidney).

The efficiency tests in 6 cases not associated with obvious myocardial failure gave fairly constant results. The blood urea was not raised, and at the most the two-hour test showed nycturia, fixation at a high level, or some slight loss of power to concentrate urine. The urea concentration test gave percentages of 2 or more.

*Chlorides.*

The food is the main source of chlorides; the normal individual varies very much in the amount of chloride excreted in a given time, and it is for this reason that a chloride concentration test is not in common use. The normal figures given vary from 5 to 25 McCracken; Mosenthal, 5 grams; Rose Bradford, 10 to 15 grams. The majority of our cases on hospital diet showed an excretion of 4–8 grams.

Retention of chlorides occurs in chronic parenchymatous nephritis, but one can assume in the absence of œdema that salt is not retained to any appreciable extent. (0·6 per cent. NaCl retained in the tissues requires 100 c.cm. of water.) The primary difficulty may be inability to excrete water and consequently retention of chloride.

A strict "chloride free diet" introduces about 1·5 grams daily, an amount within the capacity of the badly-damaged kidney to excrete. Three pints of milk means from 4 to 6 grams of salt.

Unless repeated examinations are made in nephritis the estimation is not of great practical value. Without going into the difficult question of the causation of general œdema in chronic parenchymatous nephritis, one cannot assume that deficiency in chloride or water excretion is the whole explanation.

It may be of interest to note here that excessive ingestion of salt has caused œdema. J. H. Bryant[7] reported a case of œdema of feet and legs in a man of 40 years taking 40 grams daily. The urine contained 1·86 per cent. chlorides (about three times the normal figure), and with restriction of intake the percentage fell to 0·98 per cent., with disappearance of the dropsy.

*Phenolsulphonephthalein Test.*

It is claimed for this test that it is superior to the other dyes hitherto in use in that an exact mathematical figure is obtained by the colorimetric method, further that the curve of excretion is, unlike that of methylene blue, monocyclic. The colour produced by the interaction of dye and urinary pigments is difficult to compare with the standard. Moreover, observers differ in their appreciation of intensity of colour.

We have found, as others have determined, that in a general sense it affords evidence of renal deficiency ; greatly diminished values in interstitial nephritis, high values in arterio-sclerotic kidney, low values in acute and chronic parenchymatous nephritis. The results do not run parallel with the urea concentration test. Weiss considers that when it is sufficiently abnormal to be positive it is by then associated with fairly definite physical signs. Leathes says, "Abel's test appears to perform more than it could possibly promise, and is capable of no interpretation." It certainly cannot be measured with such accuracy as to allow of an increase of diet in the event of a 5 per cent. or 10 per cent. rise in excretion.

Geraghty[8] reports on the results obtained with the test in 60 cases of urinary obstruction. As a rule the test demonstrated the greatest impairment of function in cases which had a large residual urine and had not been leading a catheter life. If the excretion was below 20 per cent., and the time of the first appearance of the dye beyond 25 minutes, the operation should be postponed without regard to the clinical condition. If with treatment the output remained low but constant, the suggestion is that the kidney has undergone chronic interstitial changes.

In 40 cases of renal infection, whether uni- or bilateral, the diseased organ was correctly indicated ; in all cases of tuberculous kidney the excretion of the drug gave a correct index of the renal function.

*Albuminuria.*

The degree of albuminuria is a useful indication of the type of kidney, the largest amounts being found in parenchymatous nephritis, variable amounts in acute and subacute nephritis, and in passive congestion down to the classical trace in renal cirrhosis.

MacLean[9] considers that neither albuminuria nor casts are of much value in estimating renal efficiency. This opinion is based on an examination of 50,000 cases. He found albuminuria in 5·62 per cent., gross in 2·55 per cent. ; albumen with casts in 1·88 per cent. (936 cases), in moderate numbers 1·1 per cent. (550 cases). He concluded that at least 550 (1·1 per cent.) of the Active Service portion of the Army consisted of men whose kidneys are inefficient. However, the finding of albuminuria will always mean a detailed clinical examination supplemented by other tests, whatever may be the percentage of apparently fit men who have albuminuria. The effect of exercise in increasing the number of cases with albuminuria was noted in the above investigation, but clinical experience does not yet permit us to say, for example, how long a case of parenchymatous nephritis has had albuminuria before seeking medical aid. It is not unusual for the supervention of œdema to call the patient's attention to his condition.

In conclusion, the problem is not that there are insufficient tests ; there are too many, several not worth employing. As a routine procedure we would lay the greatest stress on (1) clinical examination, (2) the two-hour test to estimate the quantity, day and night ratio, (3) the urea concentration, (4) blood urea examinations in acute cases and in interstitial nephritis.

REFERENCES.

1 Lichtwitz, *Praxis der Nierenkrankheiten*, 1921.
2 Mosenthal, *Med. Clin. N. Amer.*, 1920, iv. 209.
3 Newburgh, *Arch. Int. Med.*, 1921, xxviii. 1.
4 MacLean, *Brit. M. J.*, 1921, ii. 426.
5 Dobson, *Brit. M. J.*, 1921, i. 289.
6 MacAdam, *Brit. M. J.*, 1921, ii. 430.
7 Bryant, J. H., *Practitioner*, 1905, lxxv. 168.
8 Geraghty, *Tr. Am. Ass. Genito-Urin. Surg.*, 1910, v. 59.
9 MacLean, *Report No.* 43 *Med. Res. Com.*, 1919.

*Normal.*

$$\frac{D}{N} = \frac{798 \text{ c.c.}}{182 \text{ c.c.}}$$

Total urea = 21 gms.
Total chlorides = 5·04 gms.
Total phosphates = 1·5 gms. (approx.).

Specific gravity ●———————●

Urea per cent. ●- - - - - - - -●

Chlorides ● . – . – . – . ●

Phosphates ●═══════●

*Normal.*

$$\frac{D}{N} = \frac{1357 \text{ c.c.}}{330 \text{ c.c.}}$$

Urea, 25·5 gms.

$$\frac{D}{N} = \frac{938 \text{ c.c.}}{270 \text{ c.c.}}$$

Urea, 25·5 gms.

*Chronic Interstitial Nephritis.* Age 48.

7. IV. 21 15. IV. 21.

$$\frac{D}{N} = \frac{812 \text{ c.c.}}{672 \text{ c.c.}}$$

$$\frac{D}{N} = \frac{616 \text{ c.c.}}{560 \text{ c.c.}}$$

B.P. 210–130. Urine, F. and albumen, granular cast.
Blood urea, 121 mg. per 100 c.c.
Urea Concn. test, 1·36 per cent. and 1·41 per cent.
P.S.P. test < 20 per cent.
Total chlorides, 4·6 gms., 7. N. 21.

*Arterio-Sclerotic Kidney.*
(Essential hypertension.) Age 38.

Male. B.P. 230–140.
Cardiac hypertrophy.
Urine : No albumen, no casts.
$\frac{D}{N} = \frac{1036}{672}$. Blood urea, 20 mg. per 100 c.c.
Urea Concn. test, 2·53 per cent. and 2·75 per cent.
P.S.P. test, 68 per cent.
Chlorides. Totals for 3 successive days :—

| | | | | |
|---|---|---|---|---|
| Urine, | 1,330 c.c., | s.g. | 1,018, | 8.7 gms. |
| | 1,300 c.c., | s.g. | 1,015, | 8·4 gms. |
| | 1,225 c.c., | s.g. | 1,014, | 8·1 gms. |

---

J. O. Symes, M.D. Lond.,
*Physician to the Bristol General Hospital.*

Having used the tests of renal efficiency in both hospital and private practice for a period of four years, I propose discussing their value from a purely clinical standpoint in diagnosis, prognosis, and treatment.

Of all the tests I regard the two-hourly specific gravity test as the most useful, for not only do we ascertain whether there be a fixed high or low curve, which is of course unfavourable, but at the same time we get a rough idea of the urea elimination, the curve of which closely corresponds with that of the specific gravity. Incidentally, too, we measure the daily quantity of urine passed, and can compare this with the intake of fluid, and can compare the quantity of day and night urines.

The blood urea test is of great value in diagnosis, prognosis and treatment. The blood (5 c.c.) is withdrawn from a vein by means of an ordinary serum syringe, and when shaken up with a few crystals of potassium oxalate is ready for transmission to the laboratory.

The urea concentration test can be performed by the general practitioner. In private practice a close watch must be kept on the patient to see that the stipulated quantity of fluid is rigidly adhered to. Individual peculiarities with regard to diuresis are, I think, likely to vitiate the results.

The phenolsulphonephthalein test can only be performed by one skilled in colorimetric observations. So many factors, such as cardiac decompensation, renal congestion, and back pressure in the urinary tract, modify the result that the test is gradually being abandoned.

Renal efficiency tests have enabled us to distinguish between various clinical kidney conditions which formerly were difficult to separate. The following rough table will serve to illustrate this point.

| RENAL EFFICIENCY SATISFACTORY. | RENAL EFFICIENCY POOR. |
| --- | --- |
| Arterio-sclerotic kidney. | Contracted granular kidney. |
| Chronic parenchymatous nephritis. | Acute or subacute nephritis with œdema. |
| Albuminuria (postural, physiological, etc.). | Nephritis. |
| Eclampsia. | Nephritis of pregnancy. |

With greater certainty in our diagnosis there has come an increased possibility of making a correct prognosis, for on both practical and theoretical grounds it is certain that the outlook is better in those diseases in which the renal efficiency is not notably impaired. At present no renal efficiency test can be relied upon as an indication of impending uræmia.

A case of high blood-urea which is controlled by diet is more favourable than one which cannot be so regulated, and provided the diet be not altered repeated blood-urea examinations are of great service in measuring the progress of a case of nephritis.

Similarly in regard to treatment, the low blood-urea content of chronic parenchymatous nephritis is an inducement to adopt Epstein's high proteid diet, which is sometimes so effectual in dispersing the œdema. On the other hand, the high blood-urea value in chronic interstitial nephritis will be an indication to lower the protein value of the diet. It is worthy of note that the troublesome nycturia in this form of granular kidney is frequently relieved by the lessening of meat food.

Some minor matters of medical treatment are also dependent upon the results of our efficiency tests; for instance, with grave inefficiency one would naturally avoid the use of opium derivatives and of diuretics, whilst in arterio-sclerotic cases packs should be used with great caution. The operation of decapsulation in cases of subacute nephritis with œdema will largely depend, not only on the absence of cardio-vascular changes, but also upon the absence of any marked degree of renal inefficiency. These tests will also serve to distinguish the renal hemorrhage of chronic interstitial nephritis from hemorrhage due to other causes which may call for operative interference, and it is certain that they will be very widely adopted by surgeons before undertaking operations for nephrectomy and prostatectomy, or for the removal of other obstructions to the urinary outflow. It must be fully understood, however, that the tests of renal efficiency are only supplementary aids, and they in no way take the place of the most careful inquiry into the physical condition of the patient, or of the ordinary examination of the urine.

---

G. Hadfield, M.D. Lond.,
*Pathologist to the Bristol General Hospital.*

To be of the greatest service to the clinician efficiency tests must detect failure in an organ in its early stages, and give him facts where clinical signs have only given him suspicions. In the case of the kidney it is regrettable that the functional tests rarely do this.

All organs possess a physiological reserve power, and progressive disease, unless overwhelming in its initial onslaught, is always shadowed by a process of pathological hypertrophy. These two factors combine to make the determination of slight and moderate degrees of failure in any organ a problem of considerable fascination because of its great difficulties.

In the case of the kidney the shortcomings of all the available efficiency tests are due to the fact that no test measures the physiological reserve power. It is not until this is severely encroached upon that laboratory methods indicate functional failure. When the physiological reserve power is exhausted, and destruction of kidney tissue obvious from physical signs, the available efficiency tests are of proved service in prognosis, but only if considered in relation to clinical findings. The use of these tests will certainly be disappointing in the long run unless their short-comings are appreciated. The routine series of kidney efficiency tests is at present an imperfect diagnostic machine, and the lazy clinician will certainly lose many of the coppers he deposits in the slot of such a defective mechanism.

The problem of renal efficiency from a pathological point of view embraces investigations along three lines :—

1. The perfection of methods for the examination of the urine, blood, and cerebro-spinal fluid during life.

2. The correlation of these findings with the *post-mortem* appearances of the kidney and other organs.

3. The localisation of failure to a particular part of the kidney or to some other organ.

### Laboratory Methods.

*Urea Concentration Test.*

The urea estimation may be carried out by the accurate urease method, or by the considerably less accurate hypobromite method. The principle of the former test will be described later. The latter test should be performed, using a burette graduated in tenths of c.c.'s, and the evolved gas cooled. The volume of gas is transformed into the approximate urea percentage by reference to a table published by McLean, in which the hypobromite error for the urea is corrected by the urease method. I am indebted to Dr. O. C. M. Davis for pointing out to me that by doing a parallel estimation of the urinary ammonia by Folin's formaldehyde method, which takes under five minutes, an accurate figure can be obtained for the urinary urea by subtraction. This should be done in all cases where the precentage of urea falls between 1·9 and 2·2.

*Phenolsulphonephthalein Test.*

1 c.c. of a solution containing 0·6 mg. of this dye is injected into the deltoid, the patient is given a tumblerful of water to drink, and empties his bladder. At the end of the first and second hours after injection he passes water into two separate labelled bottles. The total amount of dye in each sample is estimated in a colorimeter of the Duboscq type. Comrie[1] points out several details requiring attention in this test :—

(*a*) Absorption will be delayed if the dye be injected into an œdematous area.

(*b*) The colour comparison must be made with a standard dissolved and made up in the same way as the solution used for injection, as the dye changes colour considerably with slight variations in reaction.

(*c*) Blood in the urine must be centrifuged out. Hæmoglobin makes the test a wide approximation only.

The total amount excreted should be 70 per cent. of the amount injected, and the first sample should contain most of the dye.

This test suffers from the disadvantage of many colour tests, the personal variation in colour sense in different individuals. It occasionally gives obviously aberrant results, usually on the side of a false low reading, but generally indicates advanced renal damage.

*Diastase Test.*

The smallest amount of urine which digests a 0·1 per cent. solution of soluble starch in 30 minutes at 38° C. is estimated by putting up a series of dilutions of urine (fresh or preserved under toluol), and adding a constant volume of starch. The figure is called the diastatic index. In other words, the test is one which measures the amount of a ferment of doubtful origin in a fluid of varying reaction on the theoretical assumption that the diseased kidney finds a difficulty in excreting it out of the blood-stream. It is not known whether it is one of the normal functions of the kidney to excrete it or not. Many clinical pathologists have

[1] *Lancet*, 1921, ii. 1,150.

rushed into this side-track of enzyme estimation where most physiologists, equipped with the same facts, would fear to tread. I have never known the test to give any help in judging kidney efficiency. It is certainly of empirical value in diagnosing pancreatic disease.

### The Estimation of Urea in Blood.

The earliest analysis of the blood in chronic Bright's disease seems to have been made by Dr. Guy Babington in a case under Bright's own care, who found as much urea in the circulating blood as in the urine ![1]

The estimation is carried out by decomposing the urea of the blood by the specific ferment urease contained in Soya beans.

A test tube is provided with a well-fitting cork and 20 mg. of well-powdered potassium oxalate introduced ; 4 c.c. (at least) of blood are withdrawn from a vein and quickly placed in the tube, which is at once corked and shaken ; 3 c.c. are accurately measured into a large test tube containing 0.4 gm. of Soya bean meal. The pipette used for measuring the blood is filled with 5 c.c. of a 0.6 per cent. solution of potassium acid phosphate, which is introduced into the tube. Four or five drops of caprylic alcohol are added, and the tube closed by a rubber cork carrying two tubes with rubber attachments, which are clamped. The mixture is incubated in a water-bath at 40–45° C. for half an hour and shaken occasionally. It is then connected at one side with a wash-bottle of 10 per cent. sulphuric acid, and at the other side with a tube containing 25 c.c. of centinormal sulphuric acid. Air is drawn through the apparatus, passing through the wash-bottle, the fermentation tube, and the centinormal acid in turn. After aspirating for five minutes 5 gm. of anhydrous

[1] Fagge and Pye Smith, *Principles and Practice of Medicine*, second edition, vol. ii., p. 622.

potassium carbonate and 5 c.c. of a saturated solution of sodium carbonate are added to the contents of the fermentation tube, and aspiration is continued for half an hour. The apparatus is disconnected, and the acid titrated against centinormal caustic soda. 0.4 c.c. are subtracted from the result to allow for the trace of ammonia evolved from the meal. Each 1 c.c. of acid neutralised corresponds to 10 mg. of urea.

The following sources of error should be observed :—

1. The beans used should be capable of germination.

2. The meal should be a fine powder ground in a powder-mill and sifted on a wire sieve of fine mesh.

3. Its activity should be tested from time to time by controlling it against a solution of known urea content.

4. The acid and alkali should be very accurately standardised, and the same indicator used in the standardisation as is used in the titration. The most suitable indicator is methyl red. Phenolphthalein is inaccurate for this reaction.

5. There should be a long roomy glass connection between each tube to prevent acid or alkali being aspirated into the fermentation tube or acid tube, and care should be taken when adding the alkali that none touches the delivery tubes.

By this method the upper normal limit for the urea in blood is 40 mg. per 100 c.c. This figure varies physiologically with the protein in the diet, and tends to rise with increasing age. Thus the blood should be taken after a night's fast, and a slight increase in a child or young adult given greater weight than a similar increase in a middle-aged man.

Under physiological conditions the urea of the blood varies, like all its chemical constituents, within very narrow limits. A definite increase is a sign of great value and generally of grave import. It signifies extensive kidney damage or impending total failure of some other organ, especially the heart, and although the estimation gives little information in cases of slight renal damage, it is of great service in arriving at a diagnosis of suspected impending uræmia and in the prognosis of all forms of chronic nephritis.

By the urease method figures between 90 mg. and 120 mg. are obtained in cases totally incapacitated by kidney disease —generally the contracting stage of parenchymatous nephritis, or in middle-aged men with arterio-sclerosis and simultaneous arterial disease of the kidney. A figure over 100 mg. denotes impending uræmia; in declared uræmia it often reaches 175 mg. to 200 mg.

The urea in the cerebro-spinal fluid is estimated by the same method as for blood. There is no increase except in declared uræmia, when the normal figure (20 mg. to 30 mg. per 100 c.c.) is increased to 150 mg. to 300 mg.

The correlation of the results of efficiency tests during life with the *post-mortem* estimate of the amount of kidney damage is a subject of great difficulty. A little experience soon shows that there is only the most gross parallelism in an appreciable number of cases.

Several factors contribute to this lack of agreement between the two estimates. In many cases in which efficiency tests during life indicate severe functional impairment the kidney does not show the widespread destruction associated with a pure renal death, and it becomes apparent that the functional failure depends on some other factor. This is often circulatory, and either due to myocardial failure, acting adversely on a damaged kidney, or to some grave intra-renal circulatory accident, such as widespread œdema of the interstitial tissue.

The effect of a failing general or local circulation is seen in many cases of pure arterio-sclerotic kidney, and in the pale, swollen kidney of chronic parenchymatous nephritis. Much of the inefficiency of the large white kidney is vascular in origin, and the patient improves if and when his circulation improves.

The tubules in such a kidney are widely separated by œdema fluid, and their lumina obstructed by flocculated albumen and desquamated cells. The glomerular capillaries are compressed, accounting for the pallor of the organ, while the obstructed tubules and high water-content account for its increased bulk. The glomerular filtration-pressure in such a kidney must be considerably lowered, but the amount of destruction is appreciably less than is found in chronic glomerular nephritis.

Apart from the necessity of taking the circulation into serious account, several cardinal principles are to be kept in view in making the *post-mortem* assessment.

In the first place, if a lesion destroys the glomerulus the corresponding tubule will in time atrophy ; and, on the other hand, if a tubule is destroyed its glomerulus collapses and ceases to filter. A time will come, therefore, when it will be impossible to locate the initial injury.

Secondly, in any chronic nephritis as tracts of cortical tissue are destroyed the surviving tissue hypertrophies, its glomeruli enlarge, its tubules lengthen, and the tubular epithelium proliferates. It is therefore more important to gauge the extent of this hypertrophy on which the survival of the organ depends rather than the amount of actual destruction.

All progressive chronic nephritis tends to produce a uniform type of wrecked kidney, in which tracts of compressed shrunken tubules and glomeruli lying in vascular fibrous tissue form valleys between districts, often vascular in distribution, where the parenchyma shows active hypertrophy. These hypertrophied foci project on the surface as granules, which in cases of pure renal death often show changes of fatty degeneration.

The following two cases illustrate the influence of the circulatory factor in judging renal efficiency :—

**Case 1.**—A man, aged 53, was admitted to hospital for paroxysmal dyspnœa, in an attack of which he died. Clinically he closely resembled a case of uræmia. His blood urea was 23 mg. His urea concentration test gave 2.8 per cent. and 1.4 per cent. There was a heavy cloud of albumen in the urine, and many granular casts.

*Post-mortem.*—The kidneys weighed together 7¾ oz. Section showed very conspicuous arterial disease in vessels of all calibres. There was a fair amount cf fibrosis in the cortex, but compensatory hypertrophy was well established, and the surviving tissues showed little degeneration. His heart weighed 23 cz. The myocardium showed an extreme grade of fatty degeneration, and marked coronary artery disease. There was a patch of softening in the left corpus striatum.

The primary cause of death in this case was myocardial failure.

**Case 2.**—A woman, aged 53, with twelve months' history of dyspnœa, and blood pressure of 200–140 mm. Hg., died with symptoms of uræmia and general cardiac œdema. Her blood urea was 112 mg., urea concentration test 1.8 per cent. and 0.9 per cent.

*Post-mortem.*—Her kidneys weighed together 8¾ oz., and showed universal arteriolar disease. There was well-marked active hypertrophy, and nothing like the severe grade of destruction seen in cases of chronic glomerular nephritis. The heart weighed 24 oz., and showed macroscopic and microscopic fatty degeneration of a severe degree.

Here again the primary cause of death was considered to be myocardial failure.

The effect of general and local circulation applies to all cases of nephritis, because the condition is bilateral and generalised, and always at the onset, and often throughout its course, only a part of a general tissue reaction to some general infection or toxæmia which involves the circulatory organs simultaneously with the kidney.

Focal lesions of the kidneys do not produce usually severe grades of renal inadequacy. The specific kidney of subacute bacterial endocarditis, sprinkled with glomerular infarctions from bacterial emboli, may give little indication of functional damage ; and although such cases terminate with renal symptoms, the total amount of destruction found in such kidneys seems to indicate that the failing myocardium is the cause of the terminal renal inefficiency.

In examining *post-mortem* material it must always be borne in mind that the secretory epithelium of the tubules autolyses rapidly, and some degree of desquamation and swelling is often seen in normal kidneys *post-mortem.*

## Figures and Tables

**Figure f1:**
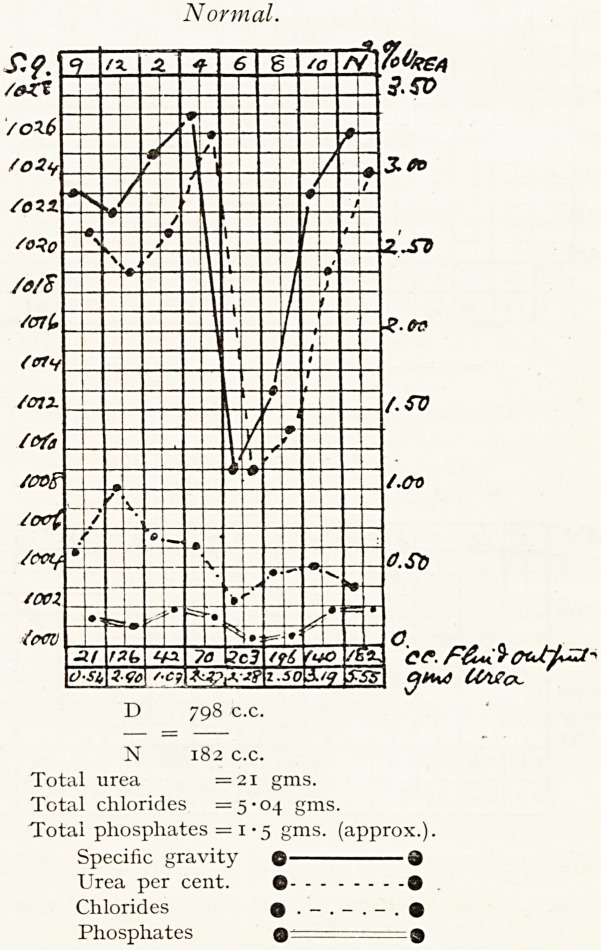


**Figure f2:**
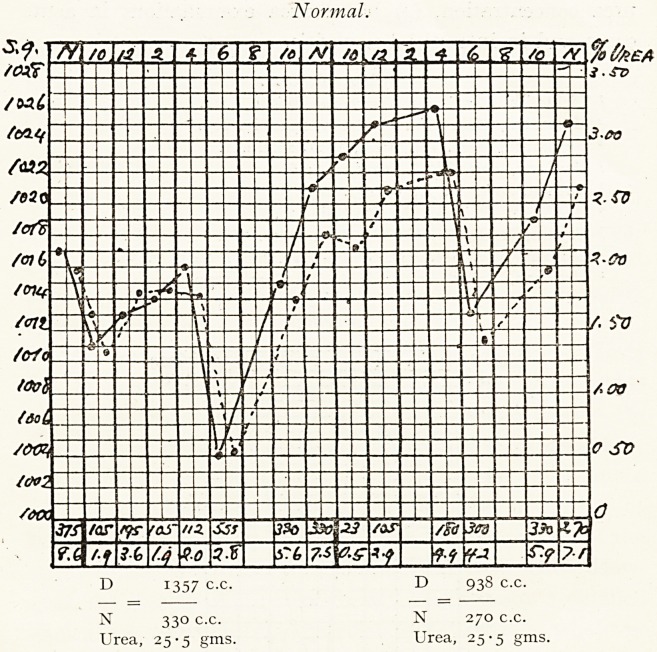


**Figure f3:**
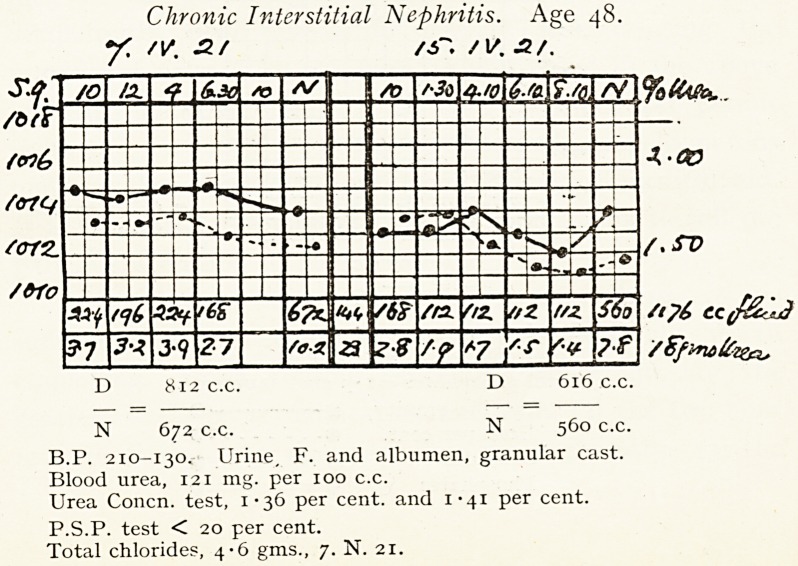


**Figure f4:**